# Checking Ablation Lines—Is Bidirectional Block Sufficient?

**DOI:** 10.19102/icrm.2025.16031

**Published:** 2025-03-15

**Authors:** Sebastian Weyand, Stephanie Löbig, Peter Seizer

**Affiliations:** 1 Medizinische Klinik II—Kardiologie und Angiologie, Ostalb-Klinikum Aalen, Aalen, Germany

**Keywords:** Ablation line gaps, atypical atrial flutter, bidirectional block, differential pacing maneuvers, slow conduction

## Abstract

This case report presents a 71-year-old man undergoing repeat ablation for atypical atrial flutter after prior pulmonary vein isolation and subsequent re-ablation involving an anterior mitral line and a posterior box. High-density mapping revealed reconnection at the left superior pulmonary vein, which was successfully re-isolated. Although bidirectional block of the anterior mitral line was confirmed via local activation time (LAT) mapping during differential pacing, burst stimulation induced atrial flutter. Further LAT mapping during flutter identified very slow conduction through a gap in the anterior mitral line. Ablation at this site restored sinus rhythm, and the arrhythmia was no longer inducible. This case highlights that bidirectional block confirmation alone may not suffice to detect gaps with slow conduction. It underscores the necessity of arrhythmia induction and mapping to reliably identify and address such gaps.

## Case presentation

A 71-year-old man presented for repeat ablation due to intermittent episodes of atypical atrial flutter. He had previously undergone pulmonary vein isolation for atrial fibrillation and a subsequent re-ablation, which involved creating an anterior mitral line and a posterior box.

Informed consent was obtained prior to the procedure.

**Figure 1:**
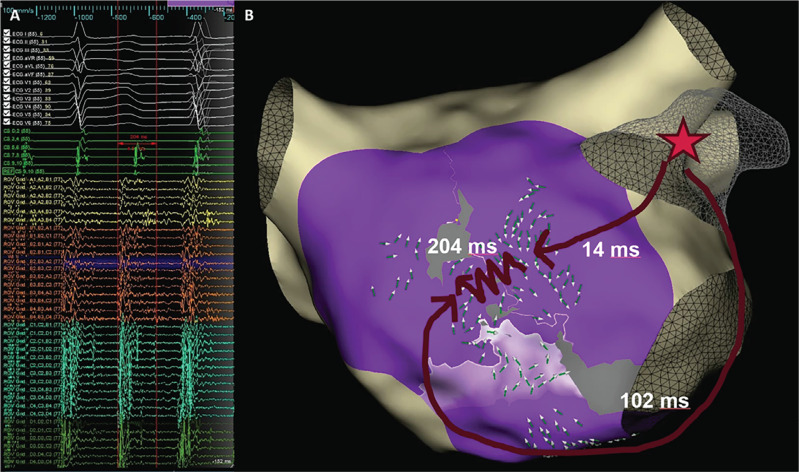
**A:** Burst stimulation induced atypical atrial flutter (cycle length, 320 ms), matching clinical tachycardia. The local activation time (LAT) map shows very slow conduction (204 ms) through a gap in the anterior mitral line. **B:** An LAT map recorded during left atrial appendage stimulation indicates bidirectional block on the anterior mitral line. The schematic suggests that an opposite-side wavefront may falsely indicate complete block despite the gap.

The repeat electrophysiological procedure was performed in sinus rhythm. High-density mapping of the left atrium revealed reconnection at the left superior pulmonary vein (LSPV). The voltage map indicated that the additional lines were blocked. After re-isolating the LSPV, local activation time (LAT) mapping was performed while pacing with a cycle length of 500 ms in the coronary sinus (CS) and during stimulation in the left atrial appendage (LAA) **(Figure 1B)** to confirm bidirectional block of the anterior mitral line. Both maps showed activation vectors converging on the line, verifying that it was bidirectionally blocked by this maneuver.

However, burst stimulation induced atypical atrial flutter with a cycle length of 320 ms, corresponding to the clinical tachycardia **(Figure 1A)**. An LAT map recorded during the flutter revealed perimitral flutter with very slow conduction through a gap in the anterior mitral line. Mapping at the gap revealed conduction through the line lasting 204 ms. Ablation at this site successfully restored sinus rhythm, and the tachycardia could not be induced again.

## Discussion

As ensuring line completeness is a class I guideline recommendation,^1^ it is typically confirmed through differential pacing maneuvers that demonstrate bidirectional block at the ablation line.^2^ This case highlights that, in the presence of very slow conduction through a gap in the anterior mitral line, demonstrating bidirectional block via stimulation maneuvers may not be sufficient to reliably exclude a gap. We hypothesize that slow conduction delays within the gap may allow wavefronts from the opposite side to arrive sooner than the slow conduction across the line, creating a false impression of a complete bidirectional block **(Figure 1B)**. This phenomenon highlights a potential limitation in relying solely on differential pacing to ensure line integrity and may explain the persistence of arrhythmias despite initial verification. This underscores the importance of attempting to induce arrhythmias to map them during flutter, thus demonstrating any gaps with very slow conduction. In addition to arrhythmia induction, further pacing maneuvers can enhance the detection of conduction gaps along the ablation line. Pacing closer to the line or dense mapping during both medial and lateral pacing can contribute to identifying slow conduction regions more effectively. Additionally, repeating the bidirectional block test after a 20–30-min interval, with pharmacological facilitation, if necessary, may improve detection reliability. A combination of these maneuvers with arrhythmia induction provides a comprehensive strategy, optimizing the available tools to locate potential gaps in ablation lines and identify relevant arrhythmia circuits. Importantly, the activation wavefronts during LAA and CS pacing may differ from those observed during induced atrial flutter, potentially concealing slow conduction that only becomes apparent with arrhythmia induction. However, arrhythmia induction, particularly through aggressive burst pacing, can reveal additional forms of atrial flutter that might not occur spontaneously as clinically relevant arrhythmias but are detectable in this setting. Therefore, induced arrhythmias should be compared with the index arrhythmias to ensure that only clinically relevant arrhythmias are targeted for treatment.

This case provides important insight into recurrence after mitral isthmus ablation: standard bidirectional block verification may not reliably exclude partial conduction gaps, especially those with slow conduction, potentially predisposing patients to arrhythmia recurrence. A more integrated approach using arrhythmia induction and additional pacing maneuvers may therefore be essential in preventing recurrence.
